# Insights of Molecular Mechanism of Xylem Development in Five Black Poplar Cultivars

**DOI:** 10.3389/fpls.2020.00620

**Published:** 2020-05-28

**Authors:** Lei Zhang, Bobin Liu, Jin Zhang, Jianjun Hu

**Affiliations:** ^1^State Key Laboratory of Tree Genetics and Breeding, Key Laboratory of Tree Breeding and Cultivation of National Forestry and Grassland Administration, Research Institute of Forestry, Chinese Academy of Forestry, Beijing, China; ^2^College of Forestry, Fujian Agriculture and Forestry University, Fuzhou, China; ^3^Biosciences Division, Oak Ridge National Laboratory, Oak Ridge, TN, United States

**Keywords:** *Populus*, developing xylem, transcriptome, cell wall, transcriptional regulation

## Abstract

Black poplar (*Populus deltoides*, *P. nigra*, and their hybrids) is the main poplar cultivars in China. It offers interesting options of large-scale biomass production for bioenergy due to its rapid growth and high yield. Poplar wood properties were associated with chemical components and physical structures during wood formation. In this study, five poplar cultivars, *P. euramericana* ‘Zhonglin46’ (Pe1), *P. euramericana* ‘Guariento’ (Pe2), *P. nigra* ‘N179’ (Pn1), *P. deltoides* ‘Danhong’ (Pd1), and *P. deltoides* ‘Nanyang’ (Pd2), were used to explore the molecular mechanism of xylem development. We analyzed the structural differences of developing xylem in the five cultivars and profiled the transcriptome-wide gene expression patterns through RNA sequencing. The cross sections of the developing xylem showed that the cell wall thickness of developed fiber in Pd1 was thickest and the number of xylem vessels of Pn1 was the least. A total of 10,331 differentially expressed genes were identified among 10 pairwise comparisons of the five cultivars, most of them were related to programmed cell death and secondary cell wall thickening. *K*-means cluster analysis and Gene Ontology enrichment analysis showed that the genes highly expressed in Pd1 were related to nucleotide decomposition, metabolic process, transferase, and microtubule cytoskeleton; whereas the genes highly expressed in Pn1 were involved in cell wall macromolecule decomposition and polysaccharide binding processes. Based on a weighted gene co-expression network analysis, a large number of candidate regulators for xylem development were identified. And their potential regulatory roles to cell wall biosynthesis genes were validated by a transient overexpression system. This study provides a set of promising candidate regulators for genetic engineering to improve feedstock and enhance biofuel conversion in the bioenergy crop *Populus*.

## Introduction

Energy issue is one of the major concerns of this century. As an important biomass energy, wood is expected to increase with the development of social economy. Biomass production as energy raw material accounts for about 14% of the world’s primary energy sources (Parikka, 2004; El Kasmioui and Ceulemans, 2012). Poplar is used as short-rotation coppice (SRC) tree and main raw materials of bioenergy because of its fast-growing, large biomass, and lower requirements for cultivation (Willebrand and Verwijst, 1993; Davis, 2008; Zhang et al., 2020). The biomass conversion rate of poplar wood is higher than that of other tree species due to its less fermentation inhibitory extract (Guerra et al., 2013).

Wood, the secondary xylem of trees, is mainly composed of cellulose, hemicellulose, and lignin. All xylem cell types first undergo secondary cell wall (SCW) thickening and experienced programmed cell death (PCD) in xylem (Courtois-Moreau et al., 2009; Zhong and Ye, 2015). While lignin content determines whether wood is used for pulp and the conversion efficiency of liquid biofuels (Wang et al., 2018). Lignin is a major phenolic polymer which is composed of 4-coumaryl alcohol (H-subunit), coniferyl alcohol (G-subunit), and sinapyl alcohol (S-subunit) (Freudenberg, 1965). Phenylalanine finally forms three monomers through the catalytic reactions of 10 enzyme families, including *PAL* (phenylalanine ammonia-lyase), *C4H* (cinnamate-4-hydroxylase), *4CL* (4-coumarate:CoA ligase), *HCT* (p-hydroxycinnamoyltransferase), *C3H* (4-coumarate 3-hydroxylase), *CCoAOMT* (caffeoyl-CoA *O*-methyltransferase), *CCR* (cinnamoyl-CoA reductase), *CAld5H* (coniferyl aldehyde 5-hydroxylase), *COMT* (caffeic acid/5-hydroxyconiferaldehyde *O*-methyltransferase), and *CAD* (cinnamyl alcohol dehydrogenase) (Freudenberg, 1965; Shi et al., 2009). And *LAC* (laccase) was involved in oxidative polymerization of lignin precursors and thus affected the process of vessel element and fiber lignification (Zhao et al., 2013). The changes of their expression can affect lignin content (Wagner et al., 2011; Lin et al., 2015; Wang et al., 2018).

The population genetic methods identified naturally occurring genetic variation for wood formation. Single-nucleotide polymorphism (SNP)-based association mapping, including quantitative trait locus (QTL) and genome-wide association studies (GWAS), has been used to identify SNPs related to wood properties in specific wood formation biosynthesis pathways in trees (Guerra et al., 2013; Zhang et al., 2018b), but only some of these associations were affiliated with genes that have *a priori* involvement in wood formation (Takata and Taniguchi, 2014; Zinkgraf et al., 2017). Transcriptional regulation is a primary mechanism that firstly responds to the environment and ultimately emits developmental signals during wood formation (Du and Groover, 2010; Zinkgraf et al., 2017; Zhang et al., 2018a). Transcriptomics has been widely used to compare and recognize specific regulatory networks in xylem development. It provides massive data for co-expression analysis, which can be used for potential gene mining and identify similar biological pathways or subject to similar regulatory pathways (D’haeseleer et al., 2000; Usadel et al., 2009). For example, Chano et al. (2017) analyzed the transcriptional profiles during the growing season in *Pinus canariensis*. Sundell et al. (2017) firstly established a high-spatial resolution transcriptome profile and revealed a gene expression module of wood formation in *P. tremula*. Subsequently, Seyfferth et al. (2018) distinguished the expression networks of ethylene-related genes in wood formation using this database.

Black poplar is widely used as the woody sources of fiber for the pulp, paper industry, biofuel production, and ecological shelter forest species in China. *P. euramericana* ‘Zhonglin46’, *P. euramericana* ‘Guariento’, *Populus nigra* ‘N179’, *Populus deltoides* ‘Danhong’, and *P. deltoides* ‘Nanyang’, are important poplar cultivars in China, and there were differences in growth and wood properties (Song et al., 2010; Yang et al., 2011; Hu et al., 2013; Zhang et al., 2020). They can represent *P. nigra*, *P. deltoides*, and their hybrids (*P. euramericana*), respectively. Wood formation mainly comes from the development of secondary xylem, which mainly refers to the deposition of lignin and thickening on the cell wall of xylem fibers and vessels (Zhang et al., 2014; Xu et al., 2017). It is great significance to explore the mechanism of cell wall formation for the study of wood formation. To obtain insights of molecular mechanism of xylem development in the five black poplar cultivars, we examined gene expression profiles of xylem and identified a large number of candidate regulators for xylem development. Three novel MYB transcription factors were identified and proved to be involved in the regulation of lignin biosynthesis. It provides new strategies and important resources for the exploration of xylem development of novel regulatory genes.

## Materials and Methods

### Plant Materials

In this study, five black poplar cultivars, *P. euramericana* ‘Zhonglin46’ (Pe1, ♀), *P. euramericana* ‘Guariento’ (Pe2, ♀), *P. nigra* ‘N179’ (Pn1,), *P. deltoides* ‘Danhong’ (Pd1, ♀), and *P. deltoides* ‘Nanyang’ (Pd2,) were used as the plant materials. Poplar trees are grown in Jiaozuo, Henan Province, China (35°14′21″N, 113°18′40″E). The stem sample was collected from breast height of the stem in an area devoid of damage. The stems were debarked in 10 cm × 20 cm region. Then, the current year’s xylem (1–2 mm) was scraped from 9-year-old trees using a sharp double-edged razor blade prior to August 2018. All the 20 samples (5 cultivars × 4 biological replicates) used for RNA sequencing (RNA-Seq) were immediately flash frozen in liquid nitrogen and then kept at −80°C until use. Then, the stem pieces, including bark, phloem, cambium, and xylem, were collected in the adjacent position by knife and reserved in formaldehyde-acetic acid-ethanol fixative (FAA) for anatomical observation.

### Light Microscopy

The stem pieces were dehydrated in a graded ethanol series and embedded in steps of 25, 50, and 75% Spurr resin and finally in 100% a full day and polymerized overnight at 60°C as described by Samuels et al. (2002). Cross section of 4-μm thick was obtained from stem by Leica M205FA. Sections were stained by 0.05% toluidine blue O (TBO) and then washed with distilled water. Finally, all the sections were examined with microscope (Zeiss). The number and diameter of vessels in each sample were measured in the same area (860 μm × 940 μm). And we measured the wall thickness of developed fibers 12–20 layers away from the cambium. All data were measured using ImageJ software.

### Illumina Sequencing and Mapping

Total RNA was isolated using the RNAprep Pure Plant Plus Kit (TIANGEN, China). Three micrograms of high-quality RNA per sample was used for the sequencing libraries preparation using NEBNext^®^ Ultra^TM^ RNA Library Prep Kit for Illumina^®^ (NEB, United States). Then, 150 bp paired-end reads were generated on an Illumina Hiseq platform. We first cleaned the raw sequences and mapped to reference genome *P. trichocarpa* v3.0^1^ using TopHat v2.0.12 (Trapnell et al., 2009). Gene expression was estimated as transcripts per million (TPM) (Li and Dewey, 2011). Sequencing data are available in NCBI SRA database (SRA number: SRP234303).

### Differential Expression Genes and Functional Analysis

To identify the differential expression genes (DEGs) between the five black poplar cultivars, we performed pair-wise comparisons (Pe1 vs Pe2, Pe1 vs Pn1, Pe1 vs Pd1, Pe1 vs Pd2, Pe2 vs Pn1, Pe2 vs Pd1, Pe2 vs Pd2, Pn1 vs Pd1, Pn1 vs Pd2, and Pd1 vs Pd2) by DESeq2 R package. The parameters used to “call a gene” between conditions was determined at a false discovery rate (FDR)-adjusted *P*-value < 0.05. We computed gene expression based on the obtained clean reads using TPM values. Gene Ontology (GO) enrichment was performed based on FDR-adjusted *P*-value < 0.05. Principal component analysis (PCA) was performed using R package.

### Clustering Analysis and Co-expression Network Construction

*K*-means clustering of the transcript expression patterns was performed using log_2_-transformed TPM in R package. Weighted gene co-expression network analysis (WGCNA) was performed according to Langfelder and Horvath (2008). The resulting network was visualized by Cytoscape 3.7.0 (Shannon et al., 2003).

### Transient Expression Assay

The coding sequence (CDS) of three novel transcription factors, *PdMYB55*, *PdMYB74*, and *PdMYB160*, were amplified from Pd1 by special primers (Supplementary Table S1). Thermal cycler program was as follows: 95°C for 5 min followed by 35 cycles of 94°C for 30 s, 58°C for 30 s, and 72°C for 50 s and a final at 72°C for 5 min. To analyze *PdMYB55*, *PdMYB74*, and *PdMYB160* transcriptional activity in yeast, the amplification products were cloned into pGBKT7 vector and transformed into the yeast strain Y2HGold containing *His3* reporter gene regulated by Gal4-responsive promoter (Liu et al., 2018). And the full-length *MYBs* were inserted in pCAMBIA2300-35S-OCS at *Acc*65I and *Sal*I sites. The empty vector was used as control. The recombinant expression vectors were introduced into *Nicotiana tabacum* leaf by transient *Agrobacterium*-mediated transformation method (Buschmann et al., 2011). After 3 days of infiltration, total RNA was extracted from infiltrated leaf region for the quantitative Real-Time PCR (qRT-PCR).

The amino acid sequences of MYB and NAC transcription factors were obtained by BLAST searchers^2^ Amino acid sequences were aligned and phylogenic analysis was performed by MEGA6.0 with the neighbor-joining method (Tamura et al., 2013).

### Quantitative Real-Time PCR

qRT-PCR was used to verify the reliability of the RNA-Seq analyses and new transcription factor (TF) roles in SCW regulation. Eight DEGs were selected for qRT-PCR analysis. Primer pairs were designed using Primer 3^3^. qRT-PCR was performed using a TB Green^®^ Premix Ex Taq^TM^ II qPCR master mix (TaKaRa, Dalian, China) according to the manufacturer’s instructions. *PtrActin* and *NtActin* were the internal controls of poplar and tobacco, respectively. The relative gene expression was calculated by the 2^–ΔΔCt^ method (Livak and Schmittgen, 2001). All experiments were performed by using three biological replicates and three technical replicates. All the primers used in this study were listed in Supplementary Table S1.

## Results

### The Microstructure of Five Cultivar Stems

**FIGURE 1:**
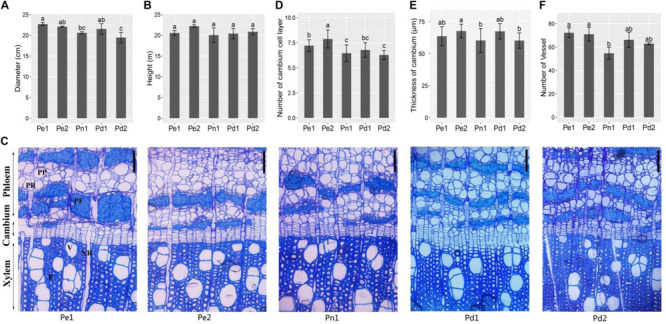
Growth traits and microscopic analysis of stem from five black poplar cultivars. The diameter **(A)** and height **(B)** of the five cultivars from 9-year-old plants. **(C)** Cross sections of phloem–xylem region from 9-year-old trees. Scale bars = 100 μm. PP, phloem parenchyma; PR, phloem ray; PF, phloem fiber; V, xylem vessel; XR, xylem ray; F, xylem fiber. Statistical analysis of the number of cambium cell layer **(D)**, thickness of cambium **(E)**, and number of vessel cells in the same region (860 μm × 940 μm, Supplementary Figure S1) **(F)**. Means ± SD from four biological replicates. Lowercase letters (a, b, and c) indicate the results of Duncan’s multiple range test (significant differences at *P* < 0.05). Pe1, *P. euramericana* ‘Zhonglin46’; Pe2, *P. euramericana* ‘Guariento’; Pn1, *P. nigra* ‘N179’; Pd1, *P. deltoides* ‘Danhong’; Pd2, *P. deltoides* ‘Nanyang’.

To find the differences of main cultivars, two *P. deltoides* (Pd1 and Pd2), one *P. nigra* (Pn1), and two hybrids (Pe1 and Pe2) were selected for analysis. Firstly, we compared the longitudinal and latitudinal growths of the five poplar cultivars from 9-year-old trees. As is shown in Figure 1A, the diameter of the two hybrids was bigger than *P. deltoides* and *P. nigra*, but no significant changes were observed in height among the five poplar cultivars (Figure 1B).

The sections of five cultivars showed that the cambial region was composed of six to eight layers cell (Figure 1C). The number of cell layer and thickness of cambium in Pe1 and Pe2 are significantly greater than Pn1 (Figures 1D,E). In xylem, there were differences in cell wall thickness between developed fibers of different varieties of xylem, among which Pd1 cell wall was the thickest (Supplementary Figure S1C). The number of vessels in the same area showed a significant difference, with Pn1 having the lowest number but the largest size of vessels (Figure 1F and Supplementary Figure S1). This indicates that the breast diameter is associated with cambium and xylem secondary growth.

### Transcriptome Sequencing and Alignment to the Reference Genome

To reveal the potential molecular mechanisms of cell wall thickening in developing xylem of the five poplar cultivars, the developing xylem was used for high-throughput RNA-Seq. A total of 10.03 billion high-quality reads were generated, of which 79.81% were successfully mapped to the *P. trichocarpa* reference genome, constituting 151 Gb of cDNA sequences. The GC content was 43.31%, and the Q30 was 93.33% (Supplementary Table S2).

**FIGURE 2:**
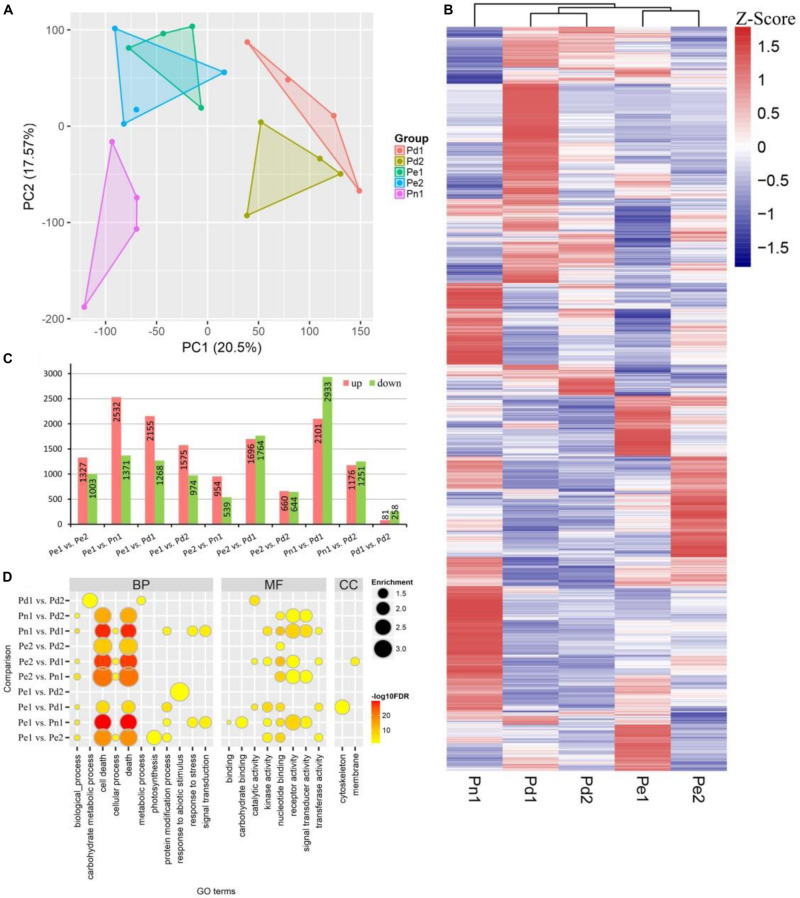
Differentially expressed genes (DEGs) in xylem of the five poplar cultivars. **(A)** Principal component analysis (PCA) of the expressed genes showing sample separation. Principal component 1 (PC1) and PC2 explaining 20.5 and 17.57% of the total variance, respectively. **(B)** Heat map of transcriptome data for the DEGs in the five poplar cultivars. **(C)** The number of upregulated and downregulated genes in pair-wise comparisons between the five cultivars. **(D)** Gene Ontology (GO) enrichment analysis of genes in different comparisons. Node color represents -log_10_ transformed false discovery rate (FDR) corrected *P*-value. Node size represents rich factor. Full list of enriched GO terms was shown in Supplementary Table S3.

A principal component analysis (PCA) plot of the whole data set revealed a sequential order of the different samples. The results showed that the five cultivars were divided into three clusters, and the biological replicates were projected closely. Two *P. deltoides* Pd1 and Pd2 were clustered together, hybrids Pe1 was clustered close to Pe2 in the middle of *P. deltoides* and *P. nigra*, highlighting the genetic relationship of five cultivars (Figure 2A).

### Analysis and Functional Annotation of Differentially Expressed Genes

To identify the global transcriptional changes in varieties, we performed a pair-wise comparison with 10 comparable groups. In total, 10,331 differentially expressed genes (DEGs) were identified (Figure 2B). The largest DEG set was identified in comparison “Pn1 vs Pd1” (a total of 5,034 DEGs, including 2,101 upregulated and 2,933 downregulated genes), suggesting the difference of *P. nigra* and *P. deltoides* ‘Danhong’. In contrast, the smallest DEG set was identified in comparison “Pd1 vs Pd2” (a total of 339 DEGs, including 81 upregulated and 258 downregulated genes) (Figure 2C).

To further characterize the biological role of DEGs, GO enrichment analysis was performed. The significant GO terms of the DEGs were classified into three major categories: 100 terms of biological process (BP), 65 terms of molecular function (MF), and seven terms of cellular component (CC) (Figure 2D and Supplementary Table S3). The most enriched terms were cell death, secondary cell wall, and lignin biosynthesis. In the BP category, subcategories of “apoptosis” (GO:0006915), “cell death” (GO:0008219), and “programmed cell death” (GO:0012501) were significantly enriched. The DEGs of “Pe1 vs Pd2” were enriched in “response to abiotic stimulus process” (GO:0009628), suggesting that there are differences in abiotic stress between the two cultivars. The GO terms “carbohydrate metabolic process” (GO:0005975) and “metabolic process” (GO:0008152) were specifically enriched in “Pd1 vs Pd2” DEGs. In the MF category, terms “ADP binding” (GO:0043531) and “receptor activity” (GO:0004872) were significantly enriched. The GO terms “nucleotide binding” (GO:0000166), “receptor activity” (GO:0004872), and “signal transducer activity” (GO:0004871) were mainly enriched in comparisons Pn1 with four other varieties. In the CC category, DEGs of “Pe1 vs Pd1” primarily belonged to “microtubule” (GO:0005874) and “cytoskeleton” (GO:0015630). “Pe2 vs Pd1” was significantly enriched in GO term “membrane” (GO:0016020).

### *K*-Means Cluster of Five Cultivars

**FIGURE 3:**
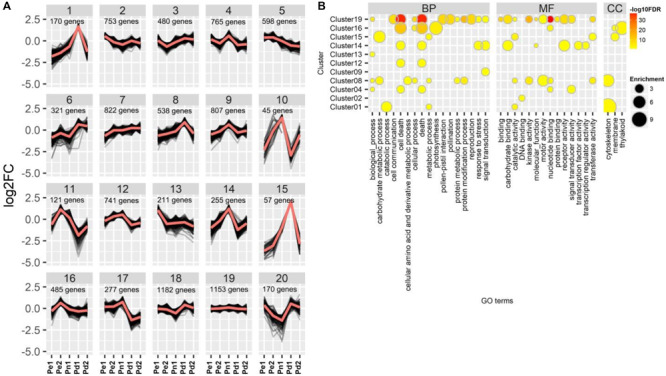
*K*-means clustering and Gene Ontology (GO) classification of differentially expressed genes (DEGs). **(A)** Gene expression profiling of clusters, showing log_2_ of fold change variations among the five poplar cultivars. **(B)** GO enrichment analysis of genes in different clusters. Node color represents −log_10_ transformed false discovery rate (FDR) corrected *P*-value. Node size represents rich factor. Full list of enriched GO terms was shown in Supplementary Table S4.

To further explore the functional diversity of DEGs from the five poplar cultivars, we performed a *K*-means clustering analysis and grouped the 10,331 DEGs into 20 clusters (Figure 3, Supplementary Data Sheet 1, and Supplementary Table S4). Three clusters (1, 8, and 15) showed a high expression level in Pd1. Genes in cluster 1 were mainly involved in “catabolic process”, “metabolic process”, “catalytic activity”, and “cytoskeleton”; genes in clusters 8 and 15 were involved in “carbohydrate metabolic process”, “membrane processes”, and “catalytic activity”. In addition, GO terms of “cellular amino acid and derivative metabolic process”, “motor activity”, and “cytoskeleton” were enriched in cluster 8. Genes in four clusters (4, 9, 10, and 14) were highly expressed in Pn1, and genes in clusters 10 and 14 showed low expression level in both Pe1 and Pd1. Genes in clusters 4 and 14 were both involved in “cell death” and “death” processes, whereas genes in cluster 14 were involved in “response to stress”, “signal transduction”, “carbohydrate binding”, and “receptor activity”. Cluster 16 which was enriched in “cell death”, “photosynthesis”, and “thylakoid” was highly expressed in Pe2. DEGs of cluster 19 did not show a difference among varieties; they were involved in fundamental categories of BP and MF.

TFs, binding to *cis*-acting elements in the promoters of target genes, as master regulators activate or repress a large number of functional genes (Gujjar et al., 2014; Yao et al., 2018). Of the 10,331 DEGs identified in this study, 671 differentially expressed TFs, including 73 bHLHs (basic helix-loop-helix), 70 MYBs, 63 NACs, and 56 ERFs (ethylene response factor), were identified in different clusters, except in clusters 10 and 15. The largest number of TFs was distributed in clusters 19 (79 TFs) and 7 (78 TFs). In addition, 10 MYBs and 5 NACs were enriched in cluster 8, which were related to cell wall biosynthesis and mainly expressed in Pd1 (Supplementary Table S5).

### Construction of Gene Co-expression Network

To obtain a comprehensive understanding of gene expression and identify novel regulatory genes during poplar wood formation, we performed a weighted gene correlation network analysis (WGCNA) using DEGs. Modules were defined as clusters of highly interconnected genes, and genes within the same module have high correlation coefficients. A total of 26 distinct modules (labeled as different colors) were identified and shown in the dendrogram (Supplementary Figure S2A). We then compared the overlapped genes between WGCNA modules and *K*-means clusters. Module turquoise (1,309 DEGs) is highly correlated with clusters 1, 8, and 15, which genes were highly expressed in Pd1 (Supplementary Figure S2B). It mainly participated in lignin, cellulose, and secondary cell wall biosynthesis, including 65% of secondary cell wall biosynthesis module, 57.6% of lignin biosynthesis module, and 48.5% of S-lignin and xylan biosynthesis module in AspWood database (Sundell et al., 2017) (Supplementary Table S6). In turquoise module, 23 putative MYB and 10 NAC genes were identified including the master switches homologous of *MYB46*, *MYB83*, *NACSECONDARY WALL THICKENING PROMOTING FACTOR 1* (*NST1*), and *SECONDARY WALL-ASSOCIATED NAC DOMAINPROTEIN 2* (*SND2*) of SCW formation (Zhang et al., 2018a) (Supplementary Figures S2D,F). Module darkturquoise is major participated in cellular component organization progress and cell wall, external encapsulating structure, and extracellular region (Supplementary Figure S2C).

**FIGURE 4:**
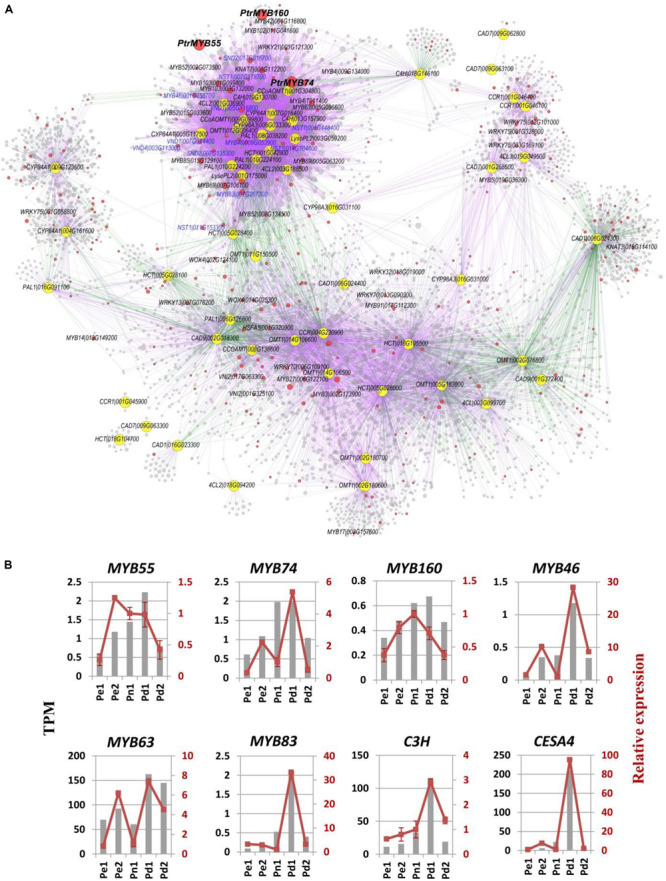
Co-expression network of lignin biosynthetic genes based on our RNA sequencing (RNA-Seq) dataset. **(A)** The subnetwork was extracted from our RNA-Seq co-expression analysis. Yellow and red nodes represent lignin biosynthetic genes and transcription factors, respectively. Purple and green edges represent positive and negative correlation, respectively. The blue letter labeled the known key regulators in the first layer and the second layer of secondary cell wall formation regulatory network (Zhang et al., 2018a). See Supplementary Table S7 for detailed node information. **(B)** Expression confirmation of eight critical genes using quantitative Real-Time PCR (qRT-PCR). Transcripts per million (TPM) values and relative expression of target genes by qRT-PCR of eight critical genes were shown. Each sample was conducted four biological replicates and four technical replicates.

Lignin synthesis pathway was regulated by three layers of regulatory network in wood plants, including MYBs, NACs, miR397a, etc. (Lu et al., 2013; Zhang et al., 2018a). To further identify potential novel regulatory genes in lignin biosynthesis, we extracted the subnetwork of lignin biosynthetic genes from our co-expression dataset (Figure 4A and Supplementary Table S7). Many known SCW regulatory TFs were identified in this subnetwork, including *MYB4*, *MYB46*, *MYB83*, *MYB102*, *NST1*, *SND2*, and *VASCULAR NAC DOMAIN 4* (*VND4*), etc. In addition, several functional unknown TFs were highly connected with those key regulators and the lignin biosynthetic genes, including four R2R3 MYB subfamily *MYB19* (Potri.009G096000), *MYB43* (Potri.011G041600), *MYB55* (Potri.014G111200), *MYB74* (Potri.015G082700), and one MYB3R4 subfamily *MYB160* (Potri.006G241700). *MYB74* directly co-expressed with genes related to lignin biosynthesis, including MYBs (*MYB46*, *MYB63*, *MYB4*, and *MYB85*), NACs (*NST1* and *SND2*), and structural genes (*PAL1*, *4CL*, *C4H*, and *CCoAOMT1*). *MYB55* indirectly co-expressed with lignin biosynthesis through a positive correlation with protein kinase and zinc finger. *MYB160* co-expressed with noyl-CoA hydratase, glutamine synthetase, and sinapine esterase, which were positive correlation with *4CL*, *C3H1*, and *CCoAMT* (Figure 4A and Supplementary Table S7). The phylogenetic relationship shows that *PtrSND2/3-B1* and *PtSND2* are the closest to *AtSND2*, *PtrVND6-A1* and *PtrVND6-B1* are the closest to *AtVND4*, *PtrVND6-C2* is the closest to *AtVND1/2*, and *PtrWND2A* is the closest to *AtNST1*. *MYB*55 and *MYB74* have the closest relationship with known SCW-associated R2R3-MYB transcription factors *PtrMYB121* and *PtoMYB170*, while *MYB160* as MYB3R4 type is the furthest from R2R3-MYB (Supplementary Figure S3).

We found 77 DEGs in our datasets that were involved in lignin biosynthesis. The highly expressed genes in Pd1 cover almost 10 enzyme families in monolignol biosynthesis and the most of *LAC*s (Supplementary Figure S4). Eight genes identified from DEG list were selected for qRT-PCR validation, which include three potential novel regulatory genes (*PdMYB55*, *PdMYB74*, and *PdMYB160*), three known MYBs (*MYB43*, *MYB63*, and *MYB83*), and two cell wall biosynthesis structural genes (*C3H* and *CesA4*). The high expression of the genes in Pd1 was consistent with RNA-seq, indicating the reliability of the RNA-seq results and the xylem of Pd1 is in active stage (Figure 4B).

### Transient Expression Assay in *Nicotiana tabacum*

**FIGURE 5:**
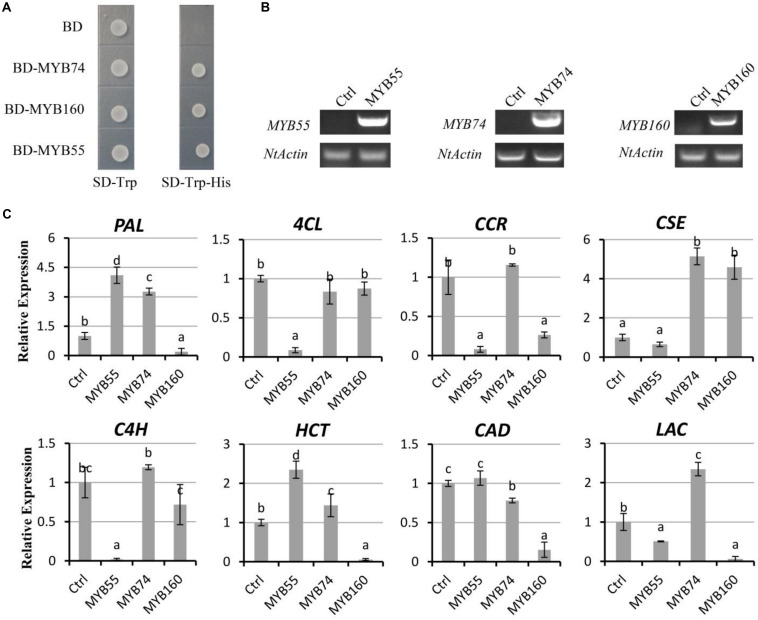
Novel transcription factors regulate the expression of the secondary wall biosynthetic genes. **(A)** Transactivation analysis of MYB in yeast. Full-length *MYB55, MYB74*, and *MYB160* were fused with the GAL4 DNA binding domain and expressed in the yeast strain Y2HGold harboring the *His3* reporter gene under the control of GAL4-responsive promoter elements. **(B)** Semiquantitative RT-PCR analysis of *MYB55*, *MYB74*, and *MYB160* expression in tobacco. **(C)** The quantitative Real-Time PCR (qRT-PCR) of structural genes in lignin biosynthesis. Error bars ± SD from three biological replicates. Lowercase letters (a, b, c, and d) indicate the results of Duncan’s multiple range test (significant differences at *P* < 0.05).

In order to verify whether these novel regulators identified in our study play potential roles in lignin biosynthesis, the three selected *MYB* genes were cloned from Pd1 and were transiently overexpressed in tobacco. Yeast cells expressing BD-MYB55, BD-MYB74, or BD-MYB160 but not BD alone grow in the absence of His (-His) on SD plates, suggesting that three *MYBs* possess the activity to promote HIS marker gene expression in yeast (Figure 5A). qRT-PCR analysis for three independent lines indicated that *PdMYB55, PdMYB74*, and *PdMYB160* can regulate the expression of lignin biosynthetic structural genes (Figures 5B,C). Similar to co-expression analysis *PdMYB74* can promote the expression of *PAL*, *CSE*, *HCT*, and *LAC*. The expression of genes in the lignin biosynthetic pathway, including *4CL*, *C4H*, *CCR*, and *CSE*, appeared strong downregulation in *PdMYB55* and *PdMYB160* transient overexpression lines compared to control plants (Figure 5C).

## Discussion

Typical poplar wood contains about 33% (vol/vol) vessel elements, 53%–55% fibers, 11–14% ray parenchyma, and about 1% axial parenchyma (Mellerowicz et al., 2001; Groover et al., 2010). In our study, there were significant differences in the number of vessels, the width of cambium region, and the xylem cell wall among five black poplar cultivars (Figure 1). Vessel, tracheary elements, transport water and soluble minerals from the roots throughout (Yamaguchi et al., 2011). Its size and number contribute to define wood density (Leal et al., 2011). Cell death is transcriptionally regulated as a part of an overall xylem maturation, which includes secondary cell wall formation (Bollhoner et al., 2012). The DEGs of Pn1 compared with the other four cultivars were enriched in cell death and death and also involved in molecular function such as kinase activity, nucleotide binding, and receptor activity (Figure 2D). And we recognized many related genes which influence the cell death and SCW of xylem vessels and fibers, such as *accelerated cell death2* (*ACD2*), *programmed cell death 4-like*, *XYLEM CYSTEINE PEPTIDASE1* (*XCP1*) and *XCP2*, *metacaspase9* (*MC9*), and *BIFUNCTIONAL NUCLEASE1* (*BFN1*) (Supplementary Table S8). VND and NST regulate vessel element and fiber differentiation (Mitsuda et al., 2005, 2007; Yamaguchi et al., 2011; Tan et al., 2018). VND6 and VND7 directly control PCD and autolysis in the element differentiation as transcriptional master switches (Escamez and Tuominen, 2014). XYLEM NAC DOMAIN1 (XND1) and VND-INTERACTING2 (VNI2) are NAC transcription factors that suppress secondary wall formation and cell death of vessel elements, suggesting they were negative regulators of xylem vessel formation (Grant et al., 2010; Yamaguchi et al., 2010). While in our study *XND1*and *VNI2* were highly expressed in *P. euramericana* and *P. deltoides*. We thought the difference of vessel development may be due to the interaction of NAC and PCD related genes, thus affecting the transport of nutrients and plant growth. We found *ERF1* (Potri.008G166200), *WRKY75* (Potri.012G101000), and disease resistance protein [CC-NBS-LRR class (Potri.T052300) and TIR-NBS-LRR class (Potri.011G014700 and Potri.019G114500)] highly expressed in Pn1 (Supplementary Figure S5), which participated in disease and defense response. And these genes were not expressed in the xylem of *P. tremula* by AspWood. The results suggest Pn1 should have stronger resistance and adaptability.

Phenotypic differences are often caused by the differential expression of genes. Only few number of DEGs (339) were identified between Pd1 and Pd2, suggesting their close relationship—they were progeny of *P. deltoides* ‘55/65’ × *P. deltoides* ‘2KEN8’ (Zhang et al., 2008; Hu et al., 2013). And all of them participated in the metabolic process, which might be the reason of radial growth differences between the two cultivars. DEGs between Pe1 and Pd1 are related with microtubule cytoskeleton (Figure 2D), which is a dynamic filamentous structure participating in nuclear and cell division, deposition of cell wall, cell expansion, organelle movement, and secretion processes in cell morphogenesis (Hussey et al., 2002).

Plant cell walls are also a source of renewable biomass for conversion to biofuels and bioproducts (Li et al., 2012). Lignin impregnate with cellulose and hemicellulose simultaneously to provide additional mechanical strength, hardness, and hydrophobicity to the secondary wall (Zhong and Ye, 2015). Zhang et al. (2018a) systematically reviewed the complex regulatory network of SCW biosynthesis, which includes a series of NAC and MYB TFs. We identified a SCW-associated module (turquoise) by WGCNA. Most genes in this module are structural genes involved in the biosynthesis of lignin and cellulose, such as *PAL*, *4CL*, *CCR*, and *CesAs*, etc. In addition, we identified a large number of transcription factors, which are known as three layers of transcription factors in the regulatory network in secondary wall thickening and lignification in wood plants, including *VND*, *SND*, and *WND* in the first layer, master switches *MYB46* and *MYB83* in the second layer, and *MYB4*, *MYB61*, and *MYB103*, etc. in the third layer (Figure 4, Supplementary Figures S2, S3). The high expression of these genes in Pd1 is related to the development state of xylem and finally leaded the thickest wall of Pd1. Evolutionary trees show the relationship between known and novel TFs (Supplementary Figure S3). For example, the deposition of lignin and thickening of secondary walls were influenced in overexpressing *PtoVNS11* transgenic poplar (Yang et al., 2015). Splicing variants of *PtrVND6-C1*^IR^ and *PtrSND1-A2*^IR^ function together to cross-regulate the VND and SND families to maintain the wood formation and plant development (Lin et al., 2017). *PtrWND2B* and *PtrWND6B* influenced the expression of SCW-associated TFs and structural genes and, concomitantly, the ectopic deposition of cellulose, xylan, and lignin (Zhong et al., 2010). *PtoMYB156* and *PtoMYB189* negatively regulate secondary cell wall biosynthesis during wood formation in poplar (Yang et al., 2017; Jiao et al., 2019). While *PtrMYB152* and *PtoMYB92* have been reported as activators of lignin biosynthesis (Wang et al., 2014; Li et al., 2015). In our study, SCW-associated modules were identified, including orthologs of *PtrSND2/3-B1*, *PtrSND1* and its target *PtrMYB021*, which influenced the thickness of secondary cell wall of xylem fiber and the content of cellulose and lignin in stem (Li et al., 2012; Wang et al., 2013). *PdMYB*55 and *PdMYB74* were clustered with *PtrMYB121*, *PtrMYB74*, and *PtoMYB170* which were identified as the downstream targets of wood-associated NAC domain TFs to influence wood formation (Zhong et al., 2011; Xu et al., 2017) (Figure 4, Supplementary Figure S3), so *PdMYB*55 and *PdMYB74* may also positively regulate lignin biosynthesis. Although *MYB160* has the furthest relationship with others, it may participate in the development of secondary wall as a member of SCW module. Those results suggested some uncharacterized NAC and MYB TFs may participate in the SCW biosynthesis.

*PdMYB55*, a homolog of *AtMYB55*, could influence the expression of key genes in lignin biosynthetic pathway in our transient expression assay. *AtMYB55*, as a brassinolide-inducible gene, participates in basal cell of mature leaves and downregulated by the Aux/IAA protein in an organ-specific manner (Nakamura et al., 2006; Schliep et al., 2010). *PtrMYB74* and *AtMYB50* as downstream genes of *NAC102* participate in the formation of secondary walls in xylem fiber and vessels (Ko et al., 2007; Zhong et al., 2011). The expression of structural genes may be upregulated by direct action in *PdMYB74*, suggesting it was a positive regulator of SCW. *PdMYB55* and *PdMYB74* are closely related in evolutionary relationship, but it is possible that their functions are not completely consistent because it regulates interaction with lignin pathway genes by protein kinase and zinc finger. *PdMYB160* belongs to c-myb-like MYB3R4 subfamily. MYB3R4 can bind to MSA motifs in promoters of B-type cyclins (CYCB) to regulate the cell cycle in *Arabidopsis* and tobacco (Haga et al., 2011; Kobayashi et al., 2015; Olszak et al., 2019). Although *PdMYB160* has transcriptional activity and represses the expression of structural genes, which may be due to the indirect effect of regulation and needs further study in the future. *PdMYB55*, *PdMYB74*, *PdMYB160*, and other SCW TFs are highly expressed in Pd1. Three MYBs and other TFs jointly regulate structural gene expression in lignin biosynthesis. These results indicate that three novel TFs are participated in the regulation of lignin biosynthetic pathway. The results of the case study prove that our dataset provides a great resource to discover novel regulators in the lignin biosynthetic pathway.

## Conclusion

Secondary cell wall biosynthesis is a biological process of producing wood, which is an important renewable material and energy raw material. The chemical structure and the content of lignin directly affect the costs of pretreatment and conversion efficiency in biofuel production from cellulosic biomass. In this study, we compared the xylem anatomical structures of five poplar cultivars in China and analyzed the transcriptome-wide gene expression profiles of developing xylem. A large number of TFs co-expressed with lignin biosynthetic genes were identified by *K*-means clustering and co-expression analysis. Furthermore, transient expression showed that *MYB55*, *MYB74*, and *MYB160* may function as novel regulators in lignin biosynthesis pathway. This study provides a useful resource for future studies seeking for the molecular mechanisms of xylem development and utilization of bioenergy.

## Data Availability Statement

The datasets generated for this study can be found in the sequencing data are available in NCBI SRA database (SRA number: SRP234303).

## Author Contributions

JZ and JH designed and conducted the experiments. LZ and BL performed the experiments. LZ and JZ conducted the data and wrote the manuscript. JZ and JH contributed to discussion and manuscript revision. All the authors were involved in the discussion of the data and approved the final manuscript.

## Conflict of Interest

The authors declare that the research was conducted in the absence of any commercial or financial relationships that could be construed as a potential conflict of interest.

## Footnotes

1 https://phytozome.jgi.doe.gov/pz/portal.html#!info?alias=Org_Ptrichocarpa

2 http://www.phytozome.com

3 http://primer3.ut.ee/

## Supplementary Material

FIGURE S1 Microscopic analysis of xylem form five black poplar cultivars. **(A)** The region for vessel count. Scale bars = 200 μm. **(B)** Average diameter of vessel. **(C)** The cell wall thickness of fiber. At least 50 cells per sample were measured. Means ± SD from four biological replicates. Lowercase letters (a, b, c, and d) indicate the results of Duncan’s multiple range test (significant differences at *P* < 0.05).

FIGURE S2 Construction of co-expression modules by WGCNA. **(A)** The cluster dendrogram of genes. Each branch in the figure represents one gene, and every color below represents one co-expression module. **(B)** The percentage of module-to-cluster in module. Node color represents the percentage in module. Node size represents gene number. **(C)** Gene Ontology (GO) enrichment analysis of genes in different modules. Node color represents -log_10_ transformed FDR corrected *P*-value. Node size represents rich factor. The heatmap of MYBs **(D)** and NACs **(E)** in module turquoise.

FIGURE S3 Phylogenetic trees of NACs **(A)** and MYBs **(B)** from different plant species by the neighbor-joining method with 1000 bootstraps. The bootstrap values are indicated as percentages at the nodes.

FIGURE S4 The differentially expressed genes related to monolignol biosynthesis **(A)** and laccase phenoloxidases **(B)** in five cultivars.

FIGURE S5 The differentially expressed genes related to defense response genes. The average TPM of four biological replicates. *ERF1*: Potri.008G166200, *WRKY75*: Potri.012G101000, *RPP4:* Potri.019G114500, *CC-NBS-LRR*: Potri. T052300 and *TIR-NBS-LRR*: Potri.011G014700.

DATA SHEET S1 Summary of all differentially expressed genes (DEGs) TPM, cluster, modules and other information.

TABLE S1 The primers used in this study.

TABLE S2 Summary of the RNA-Seq results.

TABLE S3 GO enrichment analysis of all differentially expressed genes (DEGs).

TABLE S4 GO enrichment analysis of 20 clusters.

TABLE S5 Identification of 671 transcription factors (TFs) representing 47 gene families in the 20 clusters.

TABLE S6 Common genes between turquoise module in this study and SCW-associated modules in AspWood.

TABLE S7 The genes in co-expression network of lignin biosynthetic.

TABLE S8 Differentially expressed of vessel differentiation and PCD related genes.
