# Supplementary figures and images for: Insights of Molecular Mechanism of Xylem Development in Five Black Poplar Cultivars
Source: Front Plant Sci. 2020 May 28;11:620. doi: 10.3389/fpls.2020.00620 (PMC7271880; doi:10.3389/fpls.2020.00620)

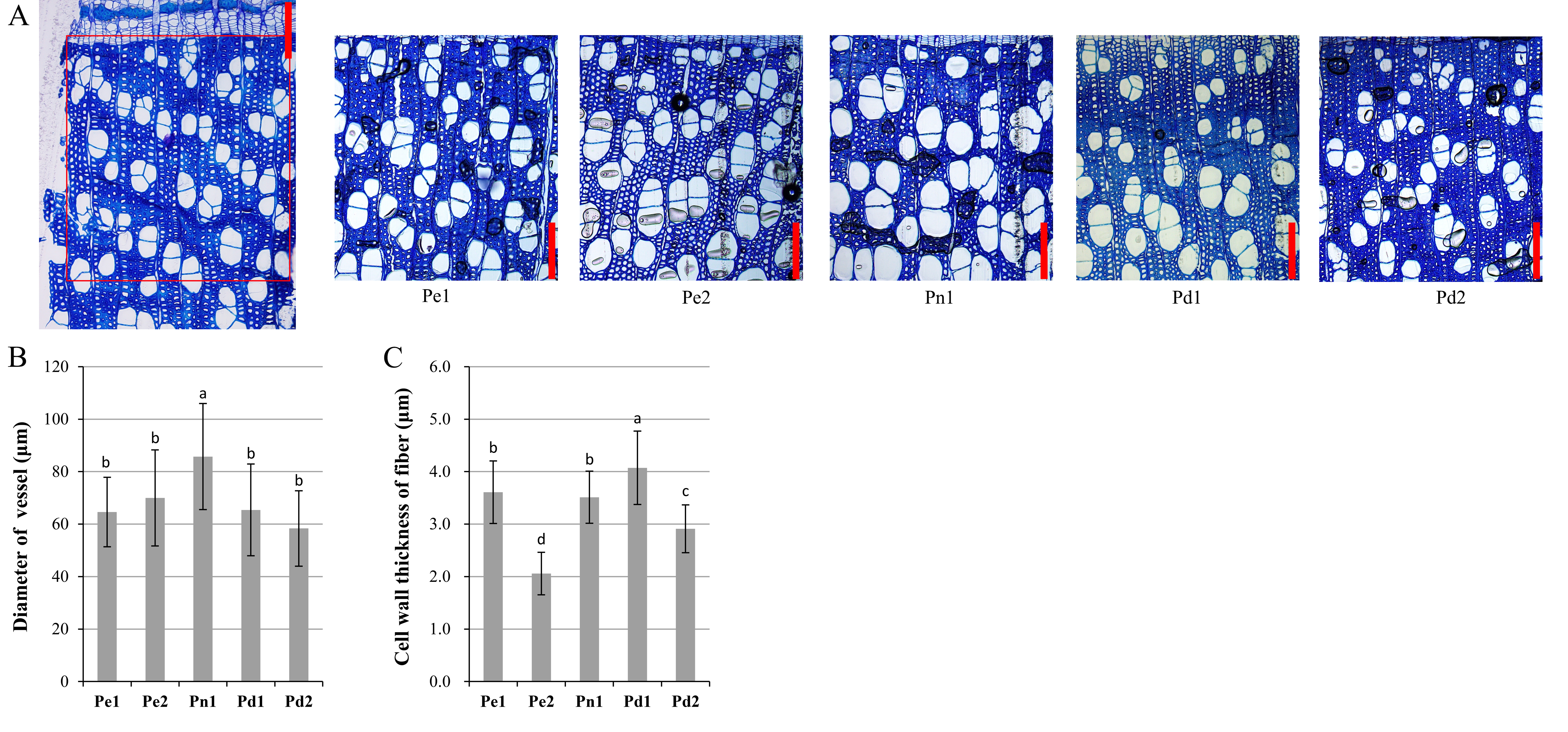

Supplement: FIGURE S1 — Microscopic analysis of xylem form five black poplar cultivars. (A) The region for vessel count. Scale bars = 200 μm. (B) Average diameter of vessel. (C) The cell wall thickness of fiber. At least 50 cells per sample were measured. Means ± SD from four biological replicates. Lowercase letters (a, b, c, and d) indicate the results of Duncan’s multiple range test (significant differences at P < 0.05). [file Image_1.JPEG]

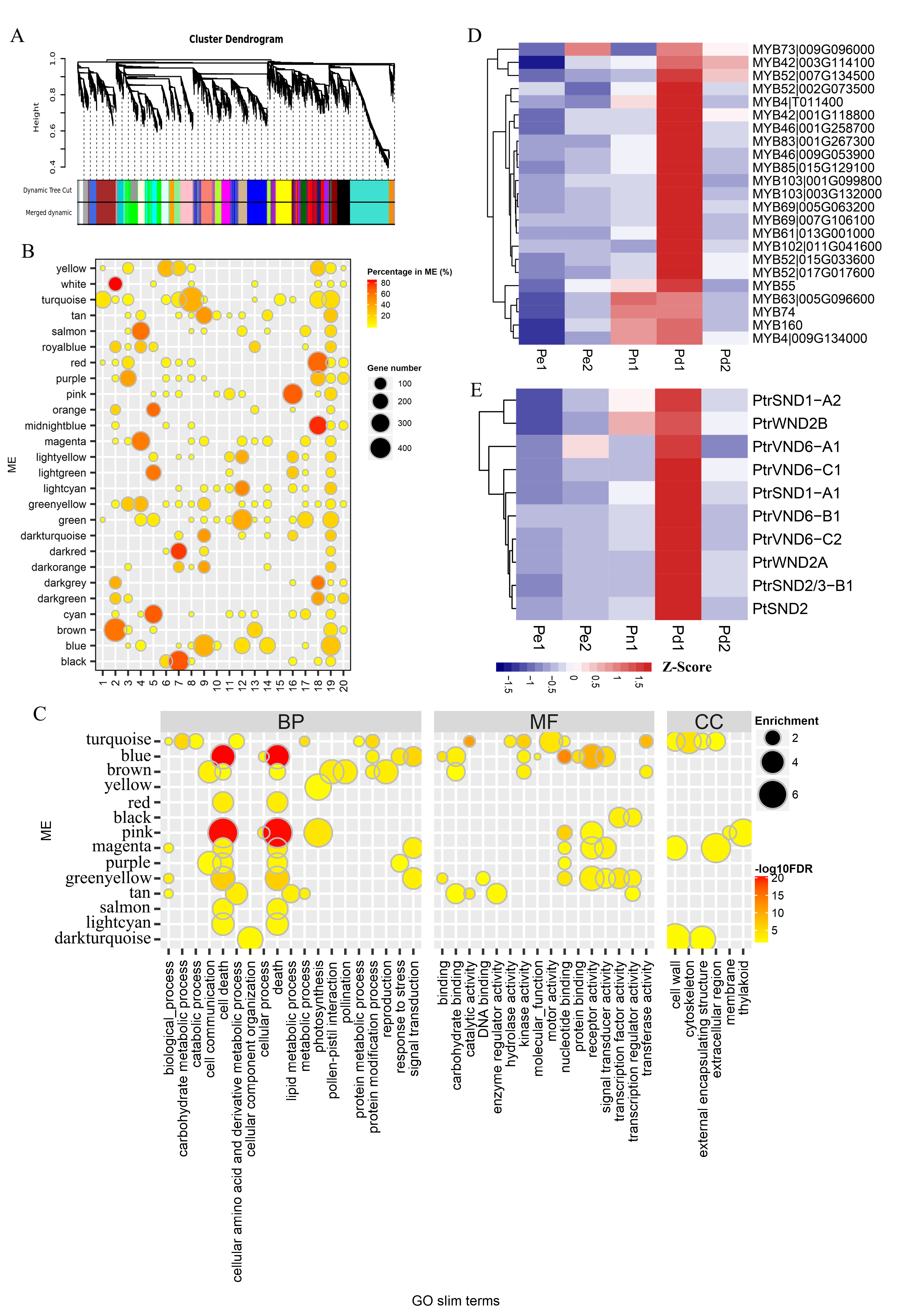

Supplement: FIGURE S2 — Construction of co-expression modules by WGCNA. (A) The cluster dendrogram of genes. Each branch in the figure represents one gene, and every color below represents one co-expression module. (B) The percentage of module-to-cluster in module. Node color represents the percentage in module. Node size represents gene number. (C) Gene Ontology (GO) enrichment analysis of genes in different modules. Node color represents -log10 transformed FDR corrected P-value. Node size represents rich factor. The heatmap of MYBs (D) and NACs (E) in module turquoise. [file Image_2.JPEG]

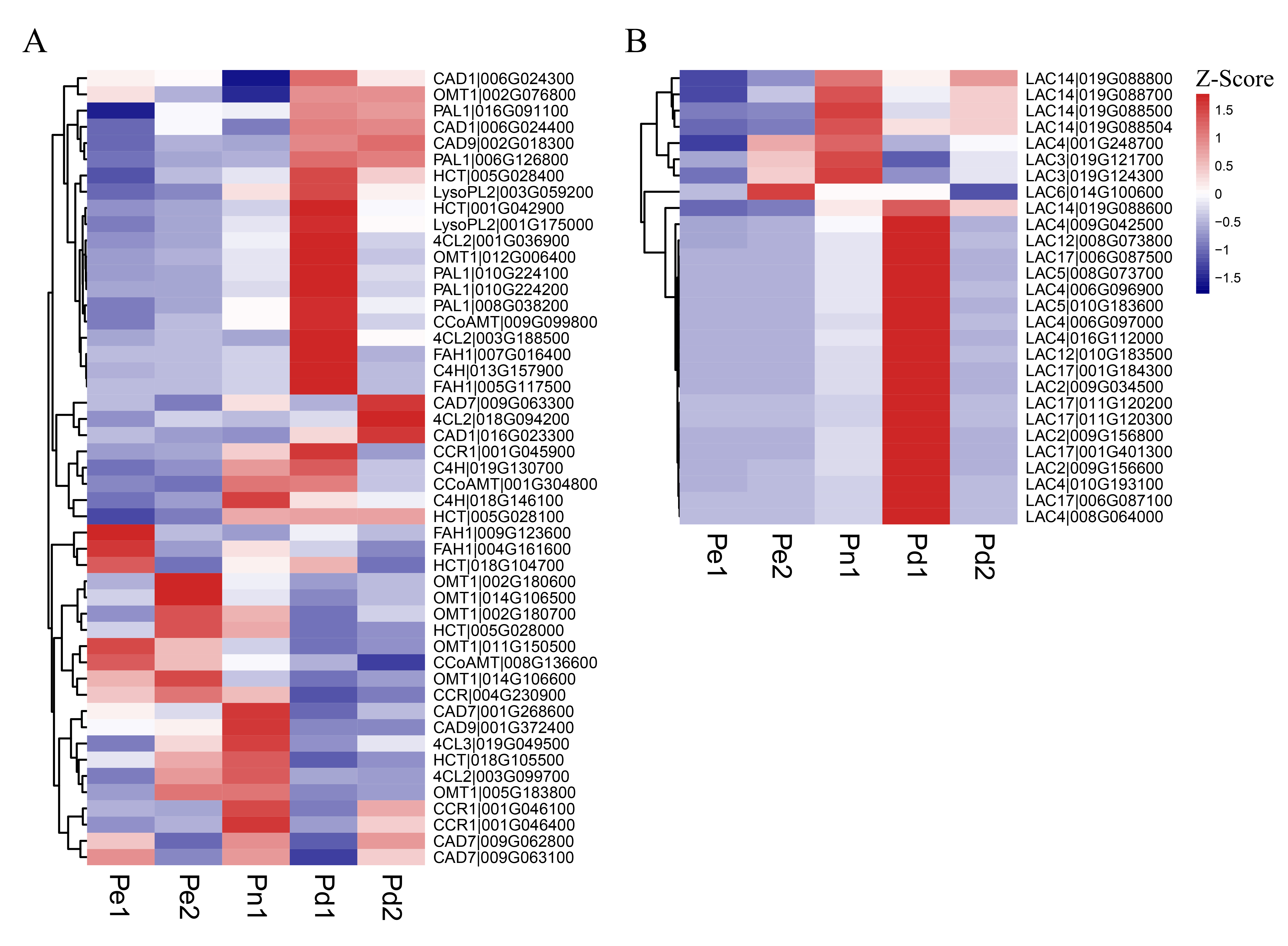

Supplement: FIGURE S4 — The differentially expressed genes related to monolignol biosynthesis (A) and laccase phenoloxidases (B) in five cultivars. [file Image_4.JPEG]

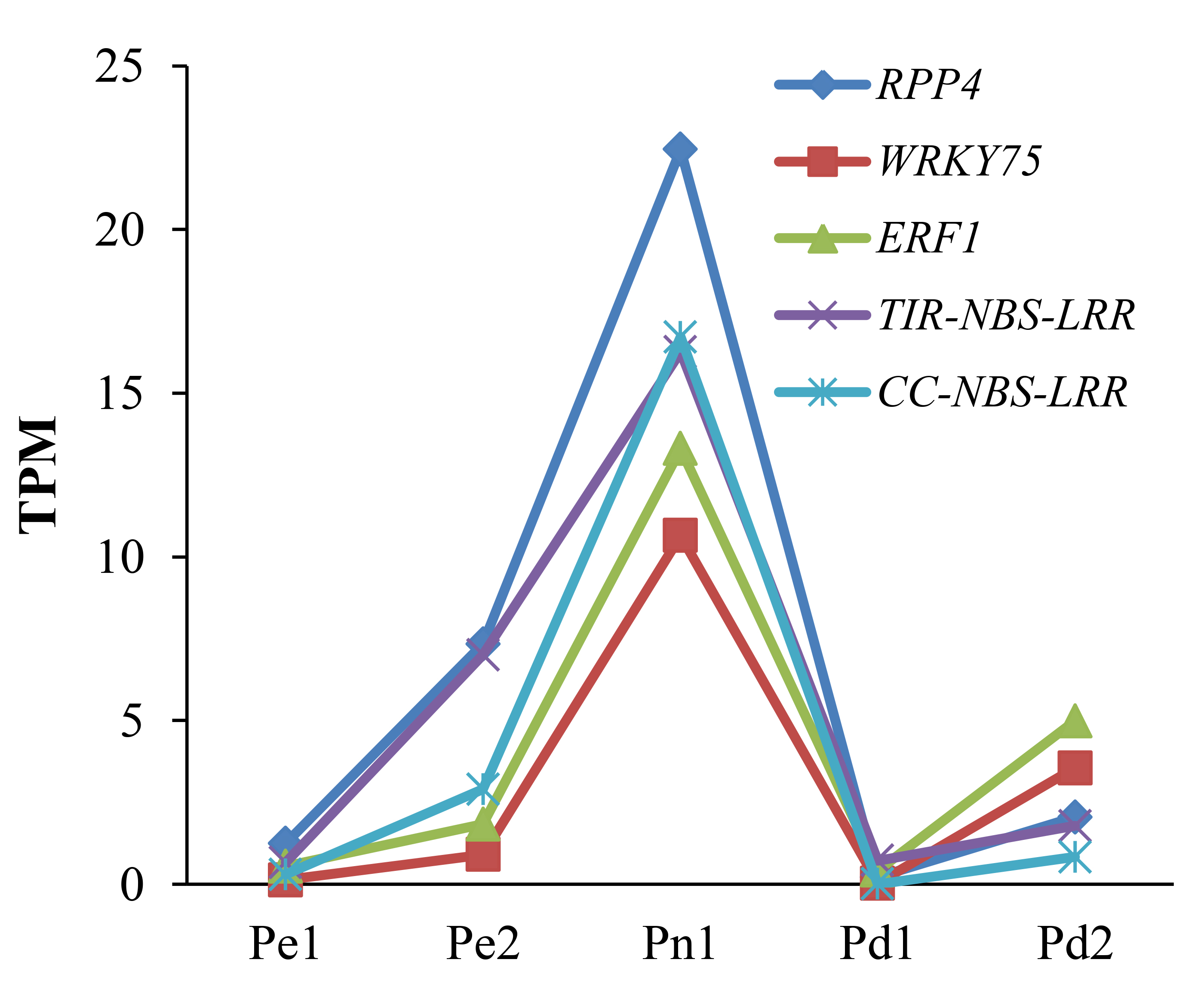

Supplement: FIGURE S5 — The differentially expressed genes related to defense response genes. The average TPM of four biological replicates. ERF1: Potri.008G166200, WRKY75: Potri.012G101000, RPP4: Potri.019G114500, CC-NBS-LRR: Potri. T052300 and TIR-NBS-LRR: Potri.011G014700. [file Image_5.JPEG]
